# Generative Sign-Description Prompts with Multi-Positive Contrastive Learning for Sign Language Recognition

**DOI:** 10.3390/s25195957

**Published:** 2025-09-24

**Authors:** Siyu Liang, Yunan Li, Wentian Xin, Huizhou Chen, Xujie Liu, Kang Liu, Qiguang Miao

**Affiliations:** 1 School of Computer Science and Technology, Xidian University, Xi’an 710071, China; syliang_233@stu.xidian.edu.cn (S.L.); yunanli@xidian.edu.cn (Y.L.); huizhouchen@stu.xidian.edu.cn (H.C.); 24031110049@stu.xidian.edu.cn (X.L.); kangliu@stu.xidian.edu.cn (K.L.); 2 Xi’an Key Laboratory of Big Data and Intelligent Vision, Xi’an 710071, China; 3 Key Laboratory of Collaborative Intelligence Systems, Ministry of Education, Xidian University, Xi’an 710071, China; 4 College of Information Science and Technology, Dalian Martime University, Dalian 116026, China; wtxin@dlmu.edu.cn

**Keywords:** sign language recognition, contrastive learning, generative large language model, modality fusion

## Abstract

While sign language combines sequential hand motions with concurrent non-manual cues (e.g., mouth shapes and head tilts), current recognition systems lack multimodal annotation methods capable of capturing their hierarchical semantics. To bridge this gap, we propose GSP-MC, the first method integrating generative large language models into sign language recognition. It leverages retrieval-augmented generation with domain-specific large language models and expert-validated corpora to produce precise multipart descriptions. A dual-encoder architecture bidirectionally aligns hierarchical skeleton features with multi-level text descriptions (global, synonym, part) through probabilistic matching. The approach combines global and part-level losses with KL divergence optimization, ensuring robust alignment across relevant text-skeleton pairs while capturing sign semantics and detailed dynamics. Experiments demonstrate state-of-the-art performance, achieving 97.1% accuracy on the Chinese SLR500 (surpassing SSRL’s 96.9%) and 97.07% on the Turkish AUTSL (exceeding SML’s 96.85%), confirming cross-lingual potential for inclusive communication technologies.

## 1. Introduction

Due to its importance in bridging communication gaps across diverse human communities, sign language recognition (SLR) has attracted substantial research interest. The advent of high-precision depth sensors, including Kinect [1] and RealSense [2], coupled with advances in pose estimation algorithms [3,4], has significantly simplified the acquisition of human joint positions in recent years. Skeleton-based SLR methods, which rely on body joint movements, have gained considerable attention due to their computational efficiency and robustness to variations in lighting conditions, viewpoints, and background noise. An increasing number of SLR works are based on the skeleton modality [5] or use skeletons as one of the multimodal inputs [6,7,8,9].

The remarkably rapid development of pre-trained generative LLMs has led to their expanding applications across various recognition domains [10,11,12], particularly in action recognition. Approaches such as GAP [13] and SpaMo [14] have demonstrated how LLMs can enhance recognition by generating fine-grained textual descriptions or interpreting spatial-motion features. Although these methods primarily employ LLMs as sophisticated text processors, their generative capabilities remain underexplored for domain-specific applications like sign language recognition. This presents several unique challenges, as sign language processing requires both linguistic precision and specialized domain knowledge that general-purpose LLMs typically lack. Therefore, potential solutions must address the fundamental tension between domain-specific motion accuracy and linguistic expressiveness in generated descriptions.

Although prompt engineering has enabled LLMs to assist in action recognition by generating auxiliary text descriptions, such approaches face unique hurdles in SLR. Sign language requires expert knowledge, as subtle variations in hand shape, motion, or expression convey different meanings. General LLMs often produce descriptions that are either overly generic or semantically inconsistent. Existing methods [13,15], designed for action recognition, struggle with inaccuracies and hallucinations in sign descriptions. This limitation calls for domain-aware prompting techniques that harmonize LLMs’ generative flexibility with the structural precision demanded by sign language. This suggests the need for new prompting paradigms that can bridge the generative capacity of LLMs with the strict descriptions of expert-defined signs.

Contrastive learning has revolutionized unsupervised representation learning in multiple domains. MoC [16] implements a momentum-based dynamic dictionary with a queue and moving-average encoder for scalable contrastive learning. SimCL [17] proposes a method for text-to-image models, treating multiple images generated from the same text prompt as positive samples. MC [18] proposes a multi-view enhanced contrastive learning method for visual representation by maximizing agreements between multi-view radiographs and their corresponding reports, solving medical imaging issues. Sign language recognition challenges contrastive learning with many-to-many relationships: One sign has multiple valid descriptions (all of which should be treated as positives), and descriptions often focus on partial actions. The single positive contrastive learning method struggles with these variable, incomplete positives, requiring new approaches that handle probabilistic alignments while preserving discrimination.

The contributions of our proposed method are as follows.

1. To the best of our knowledge, we are the first to integrate generative LLMs into SLR through our Generative Sign-description Prompts (GSP). GSP employs retrieval-augmented generation with domain-specific LLMs to produce accurate multipart sign descriptions, providing reliable textual grounding for contrastive learning.
2. We design the Multi-positive Contrastive learning (MC) approach, which combines retrieval-augmented generative descriptions from expert-validated Knowledge Bases and a novel multi-positive contrastive learning paradigm.
3. Comprehensive experiments on the Chinese SLR500 and Turkish AUTSL datasets further validate the effectiveness of our method, achieving state-of-the-art accuracy (97.1% and 97.07%, respectively). The consistent and robust performance across languages demonstrates generalization capabilities.

## 2. Related Work

### 2.1. Skeleton-Based Sign Language Recognition

Current research in skeleton-based SLR has explored several promising approaches. Self-supervised learning approaches, such as Sign-BERT [19] and BEST [7], employ masked sequence modeling and quantized pseudo-labels to learn sign representations from hand pose sequences. These methods demonstrate the potential for learning sign representations without extensive annotations, although they primarily focus on low-level motion patterns rather than linguistic semantics. Graph-based architectures like CoSign [6] utilize specialized graph convolutional networks to capture the skeletal dynamics but often neglect the global semantic relationships inherent in sign language. Meanwhile, multimodal pretraining methods such as MASA [8] integrate motion-aware autoencoders with semantic alignment to learn joint visual–linguistic representations. Despite these advances, existing methods exhibit persistent limitations by treating sign language primarily as motion sequences while neglecting its inherently structured linguistic nature. This inevitably leads to excessive dependence on data-driven motion patterns, weak integration with linguistic knowledge, and inadequate visual–semantic alignment, which are fundamental shortcomings that hinder linguistically grounded SLR systems. Thus, our work specifically addresses these gaps by multi-positive contrastive learning, aligning each sign with multiple expert-verified descriptions while maintaining cross-modal discrimination via fixed-text-encoder training. This approach learns robust visual features that absorb natural sign variations without computational overhead during inference, achieving more linguistically grounded recognition than previous methods.

### 2.2. Text-Augmented Sign Language Representation

Recent progress in combining textual and visual features for SLR predominantly adopts two distinct strategies. On the one hand, manual text encoding methods such as NLA-SLR [20] and $C^{2}ST$ [21] leverage predefined gloss vocabularies but are constrained by static description sets that fail to adequately capture execution variations and face scalability issues due to high manual annotation costs. On the other hand, generative text integration approaches, including Action-GPT [22] and GAP [13], employ LLM-based action description generation. However, these primarily target generic action recognition or vision-language tasks, leaving key SLR challenges unaddressed: hallucinated sign descriptions, lack of sign-specific linguistic constraints, and inherent difficulties in modeling simultaneous sign components (e.g., hand shape, motion, and orientation). While Sign2GPT [23] and Action-GPT [22] pioneered LLMs for sign/action description, GSP-MC introduces two novel contributions: (1) generative LLMs with RAG-enhanced domain knowledge to produce hallucination-free, discriminative sign descriptions (unlike prior LLMs used as text processors), and (2) multi-positive contrastive learning explicitly modeling the many-to-many relationships between partial skeleton motions and their valid descriptions. This approach uniquely addresses sign language’s structural semantics where existing single-positive contrastive frameworks fail.

## 3. Materials and Methods

**Figure 1:**
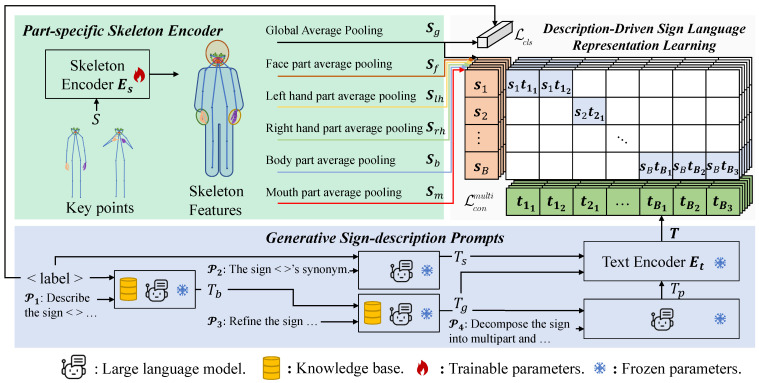
The overall architecture of the GSP-MC method. $S_{g}$ denotes global skeleton features for classification; $S_{f}$, etc., represents part-specific features for contrastive learning, and T is encoded text features. Training is guided by $L_{cls}$ and $L_{con}$ losses.

The Generative Sign-description Prompts with the multi-positive contrastive learning (GSP-MC) method augments skeleton-based sign language recognition through multimodal representation learning. As illustrated in Figure 1, the architecture comprises three elements: (1) a group-specific skeleton encoder extracting hierarchical motion features, (2) a Generative Sign-description Prompt (GSP) module producing expert-knowledge-grounded text descriptions, and (3) a multi-positive contrastive learning mechanism (MC) aligning visual and textual representations. The method maintains computational efficiency during inference using only the skeleton encoder.

The skeleton encoder $E_{s}$ extracts both the global skeleton features $S_{g}$ and the part-specific features $S_{p}$ from the input pose sequences. The GSP employs LLMs to produce descriptive texts *t*, which are subsequently encoded into text features T by the text encoder $E_{t}$. These multimodal features are then optimized through our proposed multi-positive contrastive learning approach, which enhances the model’s capacity to capture fine-grained action semantics from textual descriptions.

### 3.1. Part-Specific Skeleton Encoder

Our model processes input skeleton sequences $S\in R^{B\times 3\times N\times T}$ as input, where *B* is the batch size, 3 represents (x, y, confidence), *N* denotes the number of joints, and *T* indicates the temporal sequence length. The model predicts the labels $l\in R^{B1}$ as output.

Keypoint Selection and Grouping. Using HR-Net [4] as our skeleton estimator, we extract 87 keypoints per frame, strategically divided into five anatomical groups: 15 for the body $S_{b}$, 21 for each hand $S_{lh}$ and $S_{rh}$, 10 for the mouth $S_{m}$, and 20 for the face $S_{f}$. We provide a detailed analysis of this selection strategy in Section 4.3.3.

Skeleton Encoder. Representing the skeleton as a graph S={V,E} with joints *V* and edges *E*, we process each part *P* through layered graph convolutions.

(1) $$S_{P,l+1}=\sigma \left(D^{-\frac{1}{2}}AD^{-\frac{1}{2}}S_{P,l}\Theta_{l}\right)$$

where $D\in R^{N\times N}$ is the degree matrix. A is the adjacency matrix. $\Theta_{l}\in R^{C_{l}\times C_{l+1}}$ is the learnable parameter at layer *l*. And σ is the activation function.

Our basic block employs multiple CTR-GC blocks [24], with each block integrating a Channel-wise Topology Refinement Graph Convolution layer. We fuse channel-wise topology refinement with graph convolution to yield the final part-specific representation.

Skeleton Classification. The model optimizes a standard cross-entropy loss:

(2) $$L_{cls}=-Y\log p_{\theta }\left(S_{g}\right)$$

where Y denotes ground truth labels. $S_{g}$ represents the global skeleton features. And $p_{\theta }\left(x\right)$ is the predicted probability distribution.

### 3.2. Generative Sign-Description Prompts

**Figure 2:**
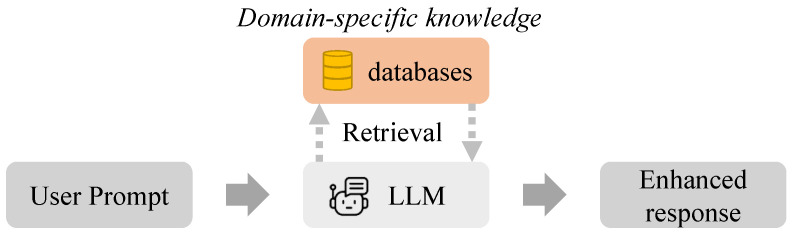
Architecture of the customized LLM for sign description generation.

The Generative Sign-description Prompts (GSPs) establish a systematic approach for generating accurate and comprehensive sign language descriptions through advanced language model techniques. As illustrated in Figure 2, the system integrates domain-specific knowledge from authoritative sources, including official sign definitions and expert textbooks. The LLM model’s DAG functionality, which we employ for retrieval-augmented generation, supports Knowledge Bases up to 1GB and individual documents up to 50 MB, providing ample capacity for our requirements.

#### 3.2.1. Expert Dataset Construction and Standardization

The method addresses the inherent challenges of professional sign language descriptions through a dual-path generation mechanism. Professional sign language definitions frequently employ substituted descriptions that create barriers for machine interpretation, manifesting as standardized manual alphabet references (“uses the ‘5’ handshape”) or cross-sign equivalencies (“identical to the sign ‘good’”). To overcome this, the system implements a rigorous standardization process where primary descriptions $T_{b}$ are generated through RAG-augmented generation anchored in authoritative sources:

(3) $$T_{b}={\mathrm{LLM}}_{\mathrm{RAG}}\left(\mathrm{label},P_{1}\right)$$

Simultaneously, the method incorporates controlled diversity through synonym variations $T_{s}$ generated by leveraging the LLM’s creative potential within designed constraints:

(4) $$T_{s}=\mathrm{LLM}\left(\mathrm{label},P_{2}\right)$$

The prompt templates $P_{1,2}$ incorporate domain-specific instruction tuning to ensure outputs maintain both high factual accuracy through expert grounding and rich linguistic diversity. For instance, a base description “palm pushes forward” might be expanded to “arm extends with palm facing outward” while preserving essential core features. This dual method effectively balances the need for standardization against the requirement for varied expressions to enhance model robustness.

#### 3.2.2. Redundancy Elimination and Multi-Part Decomposition

The method implements advanced processing to address extraneous information that is common in professional sign language materials. Through targeted prompt design $P_{4}$, the system automatically filters irrelevant content such as homophonic explanations (“‘One’ is homophonous with ‘idea’”), focusing exclusively on action-relevant descriptions. As shown in Table 1, the refinement process transforms initial outputs $T_{b}$ into complete alignment results comparing formulations $T_{g}$ while preserving expert-validated knowledge.

(5) $$T_{g}={\mathrm{LLM}}_{\mathrm{RAG}}\left(T_{b},P_{3}\right)$$

Furthermore, the system introduces innovative multi-part decomposition, automatically generating part-specific texts $T_{p}$ with corresponding anatomical annotations.

(6) $$T_{p}=\mathrm{LLM}\left(T_{g},P_{4}\right)$$

**Figure 3:**
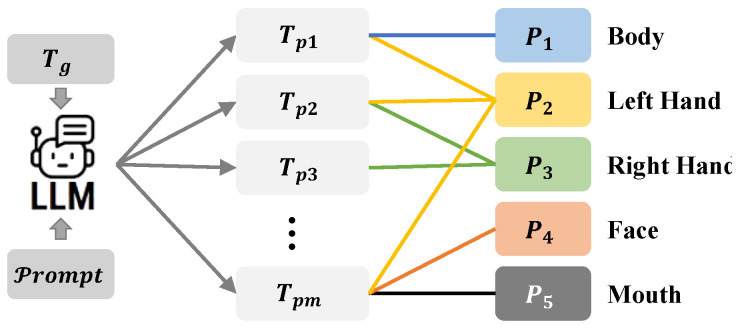
Generation of multipart texts.

The structured output $T_{p}$ pairs each text segment ($T_{p1}$, $T_{p2}$, etc.) with its corresponding sign language components. As demonstrated in Figure 3, $T_{p1}$ describes the movement involving both $part_{1}$ and $part_{2}$, while $T_{p2}$ relates to $part_{2}$ and $part_{3}$, which establishes a many-to-many mapping between text and human body parts.

#### 3.2.3. Text Encoder

The text encoding component employs the pre-trained CLIP text encoder as its backbone $E_{t}$, processing three distinct text modalities: global descriptions $T_{g}$, semantically rich synonym variants $T_{s}$, and fine-grained part-specific texts $T_{p}$. The encoding process begins with standard tokenization, followed by subsequent processing through 12 transformer blocks with multi-head self-attention mechanisms. This architecture ultimately generates context-aware hierarchical linguistic representations that are efficiently aggregated into fixed-dimensional feature vectors $T_{g}$, $T_{s}$, and $T_{p}$.

For part-specific encoding, individual body part descriptions undergo independent processing through identical architectural components, ensuring feature space consistency across all modalities. The use of frozen pre-trained weights maintains established linguistic knowledge while providing computational efficiency during training. This design choice allows the method to focus innovation on the description generation aspects while leveraging proven text encoding capabilities.

### 3.3. Description-Driven Sign Language Representation Learning

#### 3.3.1. Text-Conditioned Multi-Positive Alignment

**Figure 4:**
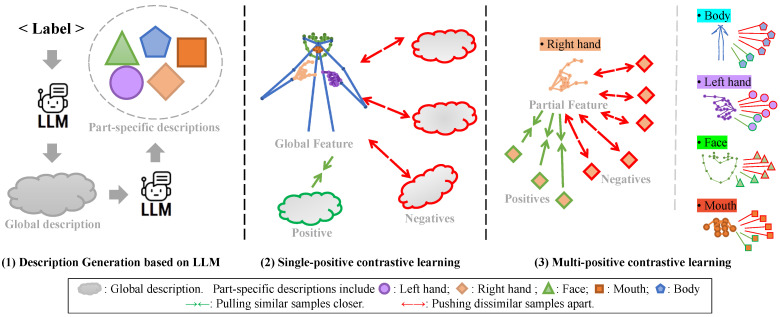
The mechanism of our Multi-positive Contrastive Learning. The process progresses from (**1**) LLM-based generation of global and part-specific descriptions, to (**2**) conventional single-positive alignment, and culminates in (**3**) our multi-positive alignment, where one skeleton feature aligns with multiple corresponding text features.

Our method uses a dual-encoder architecture. As illustrated in Figure 1, it includes a skeleton encoder $E_{s}$ and a text encoder $E_{t}$. $E_{s}$ processes skeletal motion data $S\in R^{\left(B\times 3\times N\times T\right)}$, while $E_{t}$ handles action descriptions. Unlike traditional one-hot label supervision, the proposed approach uses natural language descriptions, which provide richer supervision for skeleton classification. Our description-driven representation learning is detailed in Figure 4. The process consists of three key stages. First, as shown in Figure 4(1), our Generative Sign-description Prompt (GSP) module leverages Large Language Models (LLMs) to produce two types of descriptions for a sign: a holistic global description and several fine-grained part-specific descriptions focusing on individual body parts (e.g., hands, face, mouth).

This rich textual data enables our contrastive learning. Figure 4(2) illustrates the conventional single-positive contrastive learning scenario. Here, each skeleton sequence corresponds to one global description $T_{g}$ or synonym $T_{s}$, resulting in the number of text features *M* matching the batch size *B* (i.e., M=B). The goal is to “pull” the skeleton’s feature closer to its single positive text feature (visualized by a green arrow) while “pushing” it away from unrelated negative features (red arrows).

Our method’s primary innovation is showcased in Figure 4(3), which depicts multi-positive contrastive learning. In the part-specific scenario, multiple descriptions $T_{p}$ are generated for each skeleton, leading to M>B. Here, a single skeleton feature is aligned with multiple positive text descriptions simultaneously. As illustrated, green arrows show the feature being pulled toward all its relevant part descriptions (distinguished by shapes like diamonds for different body parts), while red arrows show repulsion from all negatives. This hierarchical representation allows the model to learn both holistic semantics and fine-grained dynamics, which is the key driver of our method’s advantage.

The dual-encoders are jointly optimized by contrasting skeleton–text pairs in two directions within the batch:

(7) $$q_{ia}^{s\to t}\left(s\right)=\frac{\exp(\mathrm{sim}\left(s_{i},t_{ij}\right)/\tau )}{\sum_{i=1}^{B}\sum_{k=1}^{m_{i}}\exp\left(\mathrm{sim}\left(s_{i},t_{ik}\right)/\tau \right)}$$

(8) $$q^{t\to s}\left(t\right)={q^{s\to t}\left(t\right)}^{T}$$

where *s*, *t* are encoded features of the skeleton and the text. $a=\sum_{b=1}^{i-1}m_{b}+j$. *j* represents the index of the *j*-th text feature within the set of *m* text features that correspond to the $s_{i}$ keypoint action feature. sim(s,t) is the cosine similarity, τ is the temperature parameter, and *B* is the batch size. In contrast to image–text pairs in CLIP [25], which are one-to-one mappings, in our proposed setting, it is possible to have more than one positive matching and actions of different categories forming negative pairs.

Given multiple candidate text features for each sign language sample, we establish a probabilistic match where at least one text feature *t* corresponds to each skeleton feature *s*. The true correspondence distribution $p\in R^{\left(B,M\right)}$ is defined as

(9) $$p_{i}=\frac{I_{match(s,t_{i})}}{\sum_{c=1}^{M}I_{\mathrm{match}(s,t_{c})}}$$

where the indicator function $I_{\mathrm{match}(s,t_{i})}$ equals 1 when *s* and $t_{i}$ share the same sign label and 0 otherwise. This formulation explicitly captures the multi-positive relationships between skeleton features and their corresponding text descriptions.

The contrastive learning objective employs KL divergence to align the predicted and true distributions:

(10) $$L_{\mathrm{con}}\left(s,t\right)=\frac{1}{2}E_{s,t∼D}\left[\mathrm{KL}\left(q^{s\to t}\left(s\right),p\right)+\mathrm{KL}\left(q^{t\to s}\left(t\right),p^{T}\right)\right]$$

where *D* is the entire dataset.

This symmetric loss function generalizes standard single-positive contrastive learning [26], where p reduces to a one-hot vector. Although related to [27], our key distinction is the explicit treatment of all texts of the same label as matched positives. Optimization brings together partial descriptions of identical signs while separating them from different signs, enhancing feature discriminability.

Our multi-positive contrastive learning fundamentally differs from standard multi-view contrastive setups. While traditional approaches like CLIP enforce a strict one-to-one correspondence between modalities (e.g., one image to one caption), sign language inherently exhibits many-to-many relationships due to execution variations and multiple valid descriptions. For instance, a single sign can be correctly described from different perspectives (holistic action, specific handshape, movement trajectory). Unlike standard contrastive learning, where each sample has exactly one positive pair, our approach models multiple valid positive pairs per sign sample through the probabilistic matching distribution in Equation (9). This explicitly captures the reality that one sign can have multiple correct textual descriptions, allowing the model to learn a more robust and comprehensive representation by pulling a single skeleton feature towards multiple text-based positive anchors in the embedding space.

#### 3.3.2. Hierarchical Part Contrastive Learning

Considering the priors of human body parts, the skeleton can be divided into multiple groups. We illustrate the overall architecture in Figure 1. We apply contrastive loss on different part features as well as global features and propose a multipart contrastive loss. The part feature could be obtained with part pooling, where joint features within the same group are aggregated to generate a part representation. More specifically, we choose the features for part feature pooling. Our part-partition strategy is five-part partition. The body is divided into five groups: left hand, right hand, face, mouth, and body. We then engage in contrastive learning by comparing the sign-relevant partial text features obtained from Section 3.2 with their corresponding body parts.

Our dual-encoder architecture employs an asymmetric training–inference design for computational efficiency. During training, the skeleton encoder learns to align its representations with multiple text descriptions through contrastive learning, effectively distilling linguistic knowledge into visual features. The text encoder acts as a teacher, guiding the skeleton encoder to develop richer semantic representations that capture both global sign meanings and part-specific dynamics. At inference time, only the skeleton encoder is deployed, as it has already internalized the semantic knowledge from the text descriptions. This design principle ensures that our method maintains the same computational efficiency as conventional skeleton-only approaches during deployment while benefiting from the superior feature representations learned through multimodal training.

The loss function of multipart contrastive loss can be represented as follows:

(11) $$L_{con}^{multi}=\frac{1}{N}\left(L_{con}\left(S_{g},T_{g}\right)+L_{con}\left(S_{g},T_{s}\right)+\sum_{i=1}^{P}L_{con}\left(S_{pi},T_{pi}\right)\right)$$

where *N* is the number of terms used to calculate the contrastive loss. The hierarchical approach offers distinct advantages by inherently respecting the compositional nature of sign language while enabling robust learning from partial observations. Through explicit modeling of part–whole relationships, it achieves superior generalization. As demonstrated in our experiments, this multi-granularity representation significantly outperforms global-only contrastive approaches while maintaining computational efficiency.

### 3.4. Composite Training Objective

Having introduced the individual components, we now formalize the complete optimization objective that jointly trains the skeleton encoder and text alignment modules. The composite training objective combines classification and contrastive losses:

(12) $$L_{total}=L_{cls}\left(S_{g}\right)+\alpha L_{con}^{multi}\left(S,T\right)$$

where $L_{cls}$ denotes the standard cross-entropy classification loss, $L_{con}^{multi}$ represents our multi-positive contrastive loss, and α is a fixed weighting parameter balancing the two objectives. The weighting parameter α controls the relative importance of semantic alignment versus classification accuracy, which we empirically set to 0.5 based on validation.

During training, the skeleton encoder processes input poses to generate $S_{g}$ through the global average pooling of all joint nodes, while $S_{p}$ is obtained by average-pooling predefined groups of related joints. Both features are projected to match the text feature dimension via separate fully connected layers. The text descriptions generated by the GSP are encoded in fixed-dimensional representations T using the text encoder. At inference time, the method utilizes only the global features of the skeleton encoder $S_{g}$ for the final prediction, ensuring no additional computational overhead compared to conventional skeleton-based approaches. During inference, the text encoder and part-specific branches are discarded, reducing the method to a single-stream skeleton encoder matching the efficiency of conventional approaches while retaining the benefits of multimodal training.

## 4. Experiments

### 4.1. Experimental Setup

#### 4.1.1. Datasets

We conduct comprehensive evaluations on two large-scale datasets.

SLR-500 [28] is a Chinese sign language dataset containing 500 vocabulary items performed by 50 native signers under controlled laboratory conditions. The dataset is divided into 87,500 training videos (35 signers) and 37,500 test videos (15 signers) for signer-independent evaluation. It provides comprehensive coverage of basic Chinese signs with natural variations in signing styles.

AUTSL [29] features 226 Turkish sign language gestures collected from 43 signers, with 28,142 training and 3742 test samples. The dataset presents unique challenges due to significant inter-signer variations and diverse recording conditions. Its medium-scale vocabulary and realistic signing variations make it valuable for robustness evaluation.

#### 4.1.2. Implementation Details

In our method, we employ HR-Net [4] to extract 87 semantically significant keypoints per frame, which our ablation studies identified as optimal for the representation of sign language. These keypoints are processed by a CTR-GCN [24] backbone with multiscale temporal convolution, preserving both spatial and temporal dynamics. The CLIP text encoder [25] processes multiple description variants, including global descriptions $T_{g}$, synonym variations $T_{s}$, and part-specific descriptions $T_{p}$, using a contrastive loss temperature parameter τ=0.1 throughout all experiments. Training protocols vary by dataset: for SLR-500 we employ 110 training epochs with batch size 120, implementing a 5-epoch linear warm-up phase followed by an initial learning rate 0.06 (gradually annealed by ×0.1 at epochs 90 and 100) and weight decay 5 × 10^−4^. The AUTSL dataset follows similar 110-epoch training, but with batch size 80, initial learning rate 0.04, and weight decay 4 × 10^−4^ while maintaining identical reduction scheduling.

Our method consists of generated content in authoritative sign language resources, including three official Chinese dictionaries [30,31,32] and the Turkish National Sign Language Dictionary [33], comprising approximately 8000 vocabulary entries (1 MB of text). For text generation, we utilize Moonshot https://platform.moonshot.cn/(accessed on 26 March 2025) AI models (v1-8K/v1-32K) to produce synonym variants, expert-verified descriptions, and part-specific texts. All implementations use PyTorch 1.8.1 running on A100-PCIE-40GB GPU hardware (manufactured by NVIDIA Corporation, Santa Clara, CA, USA).

### 4.2. Comparison with State-of-the-Art Methods

In this section, we compare our method with the existing state-of-the-art (SOTA) methods using the SLR-500 dataset. Additionally, we also conduct performance comparisons using the large-scale AUTSL sign language dataset.

#### 4.2.1. Performance Comparison on SLR-500 Dataset

Current SOTA methods exhibit several limitations that our approach addresses. While SignBERT effectively models hand gestures as visual tokens and BEST successfully applies BERT-style pre-training to triplet units, these methods predominantly focus on manual features, potentially neglecting crucial non-manual elements like facial expressions and body postures. Similarly, MASA’s motion-aware autoencoder, though powerful, faces an information bottleneck from single-positive contrastive learning.

In contrast, our method demonstrates significant advantages in several respects. First, our method benefits from the action descriptions of various body parts related to sign language in the text features, emphasizing various action features of the human body related to sign language. By incorporating these text features, our model can more comprehensively understand sign language actions, including not only hand gestures but also other important information such as facial expressions and body postures. This helps the model capture subtle differences in sign language more accurately, achieving performance comparable to or even better than SOTA methods on the SLR-500 dataset. Furthermore, our method performs well in feature representation at both the joint and bone levels, indicating that the model can effectively utilize feature information at different levels. Particularly in the four-stream setting in Table 2, our method achieved an accuracy of 97.1%, demonstrating its powerful capability to handle more complex sign language action sequences.

#### 4.2.2. Performance Comparison on AUTSL Dataset

To further verify the generalization capability of our method, we also conducted experiments on the AUTSL dataset. The AUTSL dataset is widely known for its inherent diversity and complexity, which poses even greater challenges for SLR models. As shown in Table 3, our method also demonstrated its excellent performance on the AUTSL dataset, achieving the highest Top-1 accuracy of 97.07% and Top-5 accuracy of 99.89%, representing a significant improvement over previous state-of-the-art methods.

Performance improvement can be attributed to the multi-positive contrastive learning mechanism, which provides a boost in accuracy of 1.36% by capturing inter-sign variations. Furthermore, our multimodal fusion strategy outperforms existing methods through the comprehensive integration of manual and non-manual features. These results confirm our method’s strong generalization across different sign languages and datasets, maintaining consistent performance advantages while handling AUTSL’s inherent variability challenges.

#### 4.2.3. Comprehensive Performance Analysis

The evaluation was conducted on two well-balanced datasets. The SLR500 dataset comprises 500 classes, each containing 2500 samples. The AUTSL dataset consists of 226 classes with an average of 169.6 samples per class. To provide a thorough assessment, we supplement the accuracy metric with macro-averaged precision, recall, and F1-score, as presented in Table 4.

The tight clustering of these metrics around the accuracy values (Δ < 0.6% across all metrics) confirms consistent performance across all sign categories. This metric alignment demonstrates that the high accuracy reliably reflects uniform classification capability without class-specific biases. The Top-5 accuracy further indicates robust sign identification, with correct predictions consistently appearing in the top results.

### 4.3. Ablation Study

#### 4.3.1. Combining Sign Language with LLMs

To validate the semantic quality of our generated descriptions, we conducted a detailed ablation study, with results presented in Table 5. This study indirectly assesses description quality by measuring its impact on the final recognition accuracy. The study analyzes the impact of using an unguided LLM, an expert-validated Knowledge Base (KB) alone, and our full RAG-based system against a Visual Encoder (VE)-only baseline.

Our analysis begins with a visual-only baseline, which achieves 93.85% accuracy. When we introduce descriptions generated by an unguided LLM (“+LLM only”), performance degrades by 0.28%. This confirms that general-purpose LLMs are prone to generating semantically incorrect or “hallucinated” descriptions for a specialized domain like sign language, which misleads the model.

In contrast, when we augment the Visual Encoder with text solely from our expert-validated Knowledge Base (“+KB only”), accuracy increases by 0.65%. This result establishes a crucial baseline: the expert-sourced text is semantically sound and provides valuable supervisory signals.

Finally, our full system employs Retrieval-Augmented Generation (RAG) to ground the LLM in the expert KB. This approach achieves the best performance, with a significant +1.40% improvement over the baseline. This demonstrates a powerful synergy: the KB ensures the factual correctness and semantic relevance of the generated text, effectively eliminating hallucinations, while the LLM provides linguistic diversity and rephrasing that enriches the descriptions beyond the static KB entries. The substantial performance gain serves as strong evidence that our full system generates high-quality, semantically correct, and varied descriptions that are highly beneficial for the recognition task.

#### 4.3.2. Multipart Contrastive Learning for Sign Language Recognition

Our method enhances traditional classification-based SLR by introducing a novel multipart contrastive learning mechanism. This approach is designed to effectively leverage LLM-generated knowledge (detailed action descriptions, synonyms, and expert-grounded part-specific texts), thereby fostering a deeper understanding of sign language semantics by establishing a stronger alignment between visual and textual representations.

The experimental results in Table 6 show a successive improvement in performance with each additional element introduced. The inclusion of synonym variants contributes an increase of +0.65%, while the use of optimized prompts adds another +0.88% to the accuracy. Moreover, incorporating precise part-specific descriptions delivers the most substantial improvement of +1.40%. When all these elements are fully integrated into the complete method, it achieves an impressive accuracy of 95.81%. This validates the effectiveness of our contrastive learning strategy, which successfully bridges the gap between visual and textual representation spaces, captures the nuanced differences in actions, and combines expert knowledge with the capabilities of language models.

#### 4.3.3. Keypoint Selection Analysis

The selection of keypoint combinations significantly impacts the recognition performance in skeleton-based SLR. Table 7 compares various approaches, demonstrating that our method achieves superior accuracy (93.85%), with 87 keypoints, outperforming existing combinations (27–76 keypoints) while maintaining computational efficiency.

There are limitations to previous keypoint selection strategies. SAM-SLR [37] significantly improved recognition accuracy by reducing the number of keypoints from 133 to 27. However, these methods have certain limitations in keypoint selection. SAM-SLR and MASA [8] neglect facial, lip, and other non-hand information related to sign language; although CoSign [6] collects more keypoints, it still does not cover all relevant body parts.

Through systematic examination of expert sign language descriptions, we identify 87 keypoints covering both manual (hands) and non-manual (face, mouth, body) locations commonly used in real-world signing. When combined with our multipart contrastive learning, this selection achieves a 1.4% accuracy improvement, which is significantly higher than the gains of other methods (0.16–0.95%). The results validate that complete coverage and textual grounding yield optimal recognition performance.

#### 4.3.4. Analysis of the Weighting Parameter α

**Figure 5:**
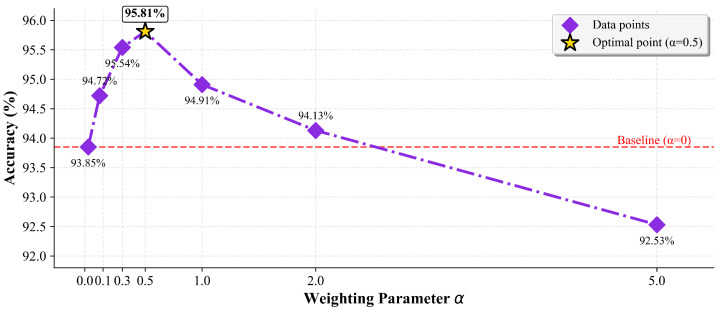
Sensitivity analysis of the weighting parameter α on the SLR-500 dataset. The baseline (α=0) uses only the classification loss.

The hyperparameter α in our composite loss function, $L_{total}=L_{cls}+\alpha \cdot L_{con}^{multi}$, balances the influence of the standard classification loss ($L_{cls}$) and our proposed multi-positive contrastive loss ($L_{con}^{multi}$). To determine its optimal value, we conducted a comprehensive sensitivity analysis on the SLR-500 dataset, with results presented in Figure 5.

The baseline model, using only $L_{cls}$ (α=0), achieved 93.85 % accuracy. As α increases from 0 to 0.5, the contrastive loss provides effective regularization, guiding the model to learn more semantically rich features, which leads to a steady performance improvement. The accuracy peaks at 95.81% when α=0.5, indicating that a balanced contribution from both loss components is optimal. However, when α increases beyond 0.5, the total loss becomes overly dominated by the contrastive objective, causing the final accuracy to decline. Notably, at a very large value like α=5.0, the performance drops to 92.53 %, even below the baseline, confirming that an excessive focus on the auxiliary contrastive task is detrimental. This analysis validates our empirical choice of α=0.5 for all main experiments.

#### 4.3.5. Analysis of Multipart Feature Contribution

To explicitly address our method’s effectiveness in capturing non-manual features (e.g., face, mouth, body), as mentioned by prior work, we conducted an ablation study on the SLR-500 dataset to quantify the contribution of different anatomical parts. We systematically removed feature groups corresponding to the face, hands, and body from our full model (VE + Multipart) and measured the impact on accuracy. The results are presented in Table 8.

The results quantify the importance of each component. As expected, hand features are the most critical, as their removal causes the largest performance drop (−1.33%). However, the experiments also provide strong quantitative evidence for the contribution of non-manual features. The removal of facial features leads to a significant decrease in accuracy of 0.70%, while the removal of body posture features results in a 0.21% drop. This confirms that our model successfully captures and leverages cues from facial expressions and body movements, moving beyond the limitations of methods that predominantly focus on hands.

**Figure 6:**
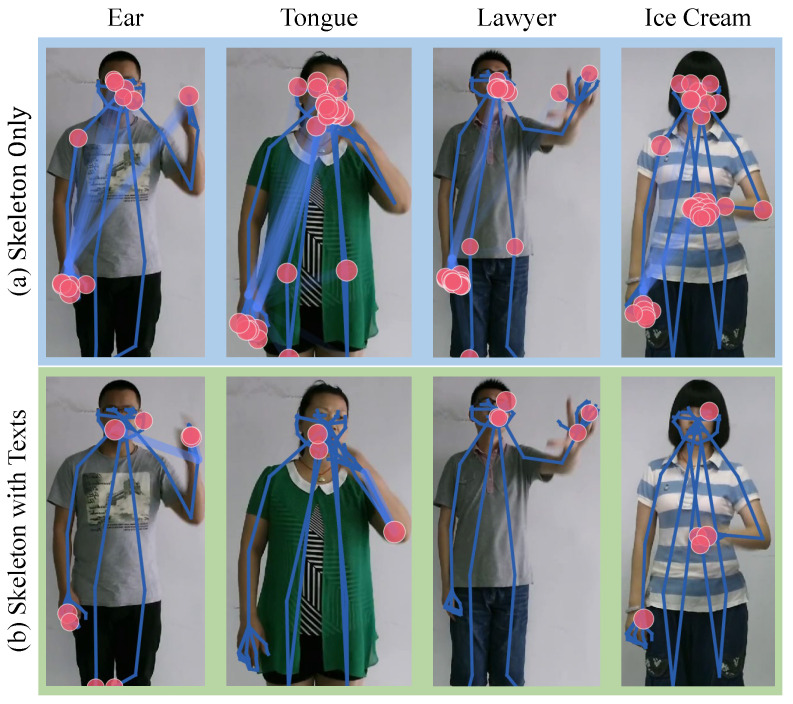
Attention Visualization Comparison: Skeleton-only (**Top**) baseline showing diffuse attention patterns. Our method’s refined attention (**Bottom**) focusing on semantically relevant joints and relationships. Color coding: pink nodes = joint attention (size indicates importance), blue links = joint relationships (width/opacity indicate strength).

Furthermore, the qualitative analysis of attention maps in Figure 6 corroborates these findings, showing that our model learns to focus on relevant facial or body regions for specific signs, unlike baselines that might over-attend to hands. Collectively, this analysis validates that our multipart framework effectively integrates both manual and non-manual information for more robust and accurate sign language recognition.

### 4.4. Visualized Analysis

To provide qualitative and deeper insights into our method’s behavior, we conduct a comprehensive visual analysis using the SLR-500 trained model. This examination reveals how the integration of textual descriptions influences both spatial attention patterns and categorical performance across diverse sign categories.

#### 4.4.1. Visualization of Human Keypoint Attention Weights

In Figure 6, we visualize the attention patterns of our skeleton encoder to analyze the impact of contrastive learning based on multipart descriptions. The attention weights are derived from channel-averaged feature maps, where joint importance is computed through normalized attention aggregation. Before incorporating text features, the model exhibits diffuse attention across multiple joints, indicating weaker semantic alignment. After applying our MC method with text guidance, the model significantly reduces attention noise, focusing more precisely on linguistically meaningful regions. For instance, in the Class “Tongue”, the model correctly emphasizes left-hand and facial joints, while the Class “Ear” shows enhanced attention to relevant inter-joint relationships. The visualization confirms that our method not only suppresses irrelevant keypoints but also strengthens the model’s ability to capture sign language semantics.

#### 4.4.2. Class-Wise Performance Gains

**Figure 7:**
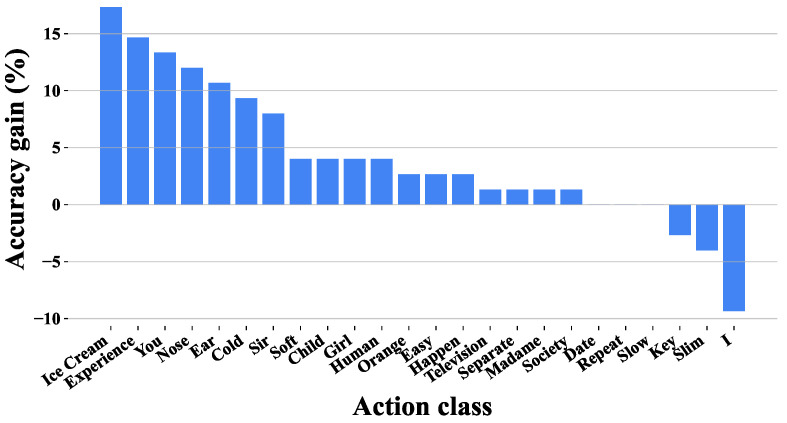
Class-wise Performance Gains.

Figure 7 presents a detailed analysis of the class-wise accuracy improvements of our GSP-MC method versus the baseline CTR-GCN model on SLR-500. The visual-text contrastive learning method produces markedly different impacts across sign categories, revealing important patterns about its operational mechanisms.

The substantial 17.33% performance gain for “ice cream” stems from its precise description specifying “bend fingers + press cheek + pinch thumb-index”, while “experience” improves by a notable 14.67% with explicit “circular motion near forehead” trajectory details. Conversely, “thick” declines by 12% due to its vague “press down” description lacking critical body part and trajectory specifics, and “I” drops by 9.33% when generated texts confuse pronouns. Recognition accuracy proves to be directly dependent on complete descriptions, particularly when specifying exact body configurations (“bend fingers”), anatomical locations (“cheek”), and motions (“circular”).

These results collectively demonstrate that our approach excels most for signs with concrete physical referents that are amenable to clear part-specific decomposition. Performance variations, both positive and negative, directly correlate with description quality. The fact that 4/5 of the classes maintained or exceeded baseline performance confirms the overall robustness of the multipart contrastive learning approach.

#### 4.4.3. Case Study: Illustrating the Many-to-Many Mapping

**Figure 8:**
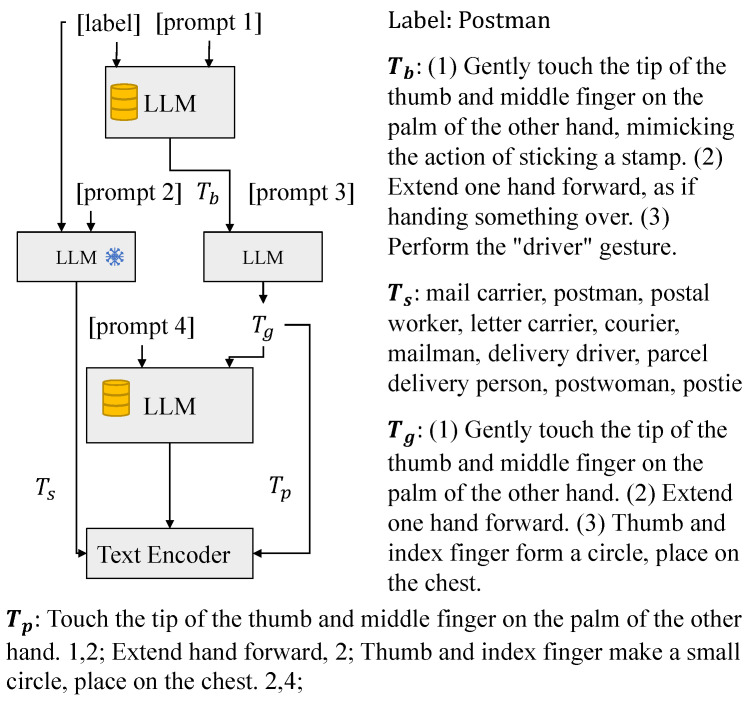
An illustration of the many-to-many mapping generated by our GSP module for the Chinese sign “Postman”. For a single sign instance, the module produces (a) a detailed global description ($T_{g}$), (b) a set of synonyms ($T_{s}$), and (c) segmented part-level descriptions ($T_{p}$). The numeric labels indicate the associated body part: 0 (unrelated), 1 (left hand), 2 (right hand), 3 (face), and 4 (body).

To illustrate the many-to-many mapping mechanism, which is central to our multi-positive contrastive learning, we present a case study of the Chinese sign “Postman” from the SLR-500 dataset. As shown in Figure 8, our GSP module generates a hierarchical set of textual descriptions for this single sign instance. This process exemplifies the one-sign-to-many-texts relationship, where a single skeleton sequence is associated with multiple positive text anchors. Specifically, these anchors include (1) a refined global description $T_{g}$, such as “(1) Gently touch the tip of the thumb and middle finger on the palm of the other hand, mimicking the action of sticking a stamp. (2) Extend one hand forward, as if handing something over. (3) Perform the “driver” gesture.”; (2) a collection of synonym variants $T_{s}$, such as “mail carrier” and “postal worker”; and (3) a set of atomic part-level descriptions $T_{p}$ segmented from $T_{g}$.

Furthermore, the part-level descriptions establish an explicit many-texts-to-many-skeleton-parts alignment. For instance, a single text segment like $T_{p1}$ (“…on the palm of the other hand.”) describes a coordinated action involving both hands and is therefore mapped to both the left-hand $S_{lh}$ and right-hand $S_{rh}$ skeleton feature groups. Conversely, a single skeletal component, such as the right-hand group $S_{r}h$, participates in multiple distinct actions described by different text segments $T_{p1}$, $T_{p2}$, and $T_{p3}$. This example illustrates how our method moves beyond a simple one-to-one paradigm. By simultaneously optimizing alignments between a single sign’s skeleton data and its rich, multi-faceted textual descriptions, the model learns a feature representation that is robust to variations and grounded in the compositional nature of sign language.

### 4.5. Efficiency and Computational Cost Analysis

To evaluate the practical viability and deployment potential of our method, we conducted a comprehensive analysis of its computational costs, comparing it against the CTR-GCN baseline. The results, including training time, GPU memory usage, inference speed, and model parameters, are summarized in Table 9.

As shown in Table 9, the training phase of our method requires more resources than the baseline. The training time is approximately 2.6 times longer, and the GPU memory usage is nearly doubled. This increased cost is attributable to three main factors: (1) the one-time, offline text generation and preprocessing using LLMs (3 h); (2) the additional forward passes required for the dual-encoder architecture; and (3) the computational demands of the multi-positive contrastive loss.

However, our central claim of efficiency pertains to the inference stage. At an average of 4.3 ms per sample, our model’s inference speed translates to a throughput of approximately 232 samples per second (fps). This high performance surpasses typical real-time requirements (e.g., 30 or 60 fps) and confirms that our method introduces no meaningful overhead compared to the baseline (4.2 ms). This efficiency is possible because the text encoder (63.5 M parameters) is discarded after training, leaving the deployed model with the same parameter count (5.6 M) as the baseline. Moreover, since the inference time scales linearly with sequence length, our model robustly maintains its suitability for practical, real-time applications.

## 5. Discussion

While our method demonstrates strong performance, a comprehensive discussion of its limitations, practical significance, and deployment challenges is warranted to contextualize the results and guide future research.

### 5.1. Limitations and Future Directions

Our study has several limitations that open avenues for future work:

1. The most significant limitation is our evaluation on isolated sign datasets. While this provides a controlled environment to validate our core approach, real-world communication involves continuous signing. Extending our method to Continuous Sign Language Recognition (CSLR) presents substantial challenges, including (a) temporal segmentation to identify sign boundaries; (b) modeling co-articulation effects, where the appearance of a sign is influenced by adjacent signs; and (c) interpreting grammatical structures conveyed over time. Future work will focus on adapting our framework for CSLR, potentially by integrating it into a sequence-to-sequence architecture (e.g., Transformer or CTC-based models) to process continuous streams on datasets like RWTH-PHOENIX-Weather [42] and CSL-Daily [43].
2. Our method’s performance is contingent on the accuracy of the upstream pose estimation model. In real-world scenarios with challenging conditions such as poor lighting, motion blur, or occlusions, the quality of the extracted skeleton data may degrade, consequently affecting recognition accuracy. Improving robustness to imperfect pose estimation is a key direction for future research.
3. The LLM-based description generation introduces a one-time computational cost for each vocabulary item. While this is an offline process and does not affect inference speed, it adds complexity to the training pipeline compared to traditional methods.
4. Although we have demonstrated success on both Chinese (CSL) and Turkish (AUTSL) sign languages, our model has not been tested on a wider range of sign languages or the vast stylistic and regional variations that exist within them. Future work should explore the model’s generalizability and potential need for fine-tuning on diverse linguistic and signer-specific data.

### 5.2. Practical Significance and Deployment Considerations

Despite these limitations, our method has significant practical implications:

1. The state-of-the-art accuracy (>97%) on large-scale isolated sign datasets makes the method suitable for immediate deployment in applications where single-sign interaction is prevalent. This includes educational software for learning sign language, assistive communication boards, and command-based control systems for smart devices.
2. By leveraging fine-grained textual descriptions, our model learns a more semantically rich representation of signs compared to methods relying solely on class labels. This could enable more nuanced applications, such as sign-to-text systems that can differentiate between visually similar but semantically distinct signs.
3. Importantly, the LLM and text encoder are only used during the training phase. During inference, our model operates solely on skeleton data, maintaining the same computational efficiency as standard skeleton-based classifiers. This makes it practical for real-time deployment on resource-constrained devices.

However, real-world deployment must address several challenges: (a) ensuring the entire pipeline, from video capture to recognition, meets real-time processing requirements; (b) building robustness to diverse signing styles, speeds, and environmental conditions; (c) ensuring seamless integration with existing accessibility infrastructure; and (d) navigating data privacy and ethical considerations related to the collection and use of sign language data.

## 6. Conclusions

This paper introduced GSP-MC, which achieves significant performance gains through three key theoretical innovations. First, the Generative Sign-description Prompts succeed where they address the semantic gap between visual features and linguistic meaning, using RAG to ground LLM outputs in expert knowledge, prevent hallucinations, and maintain descriptive richness. This provides a 0.88% improvement over raw LLM integration, demonstrating that domain-specific knowledge anchoring is essential for specialized recognition tasks.

Second, the multi-positive contrastive learning proves effective by acknowledging the inherent many-to-many relationship in sign language: one sign has multiple valid executions and descriptions. Unlike single-positive methods that force artificial one-to-one mappings, our probabilistic alignment (Equation (9)) naturally handles variation, contributing 1.40% accuracy gain. This theoretical shift from deterministic to probabilistic matching better reflects the linguistic reality of sign language.

Third, the hierarchical part-based decomposition works because sign language is compositionally structured, where meaning emerges from coordinated movements of hands, face, and body. By explicitly modeling these components (87 keypoints across five body parts), we capture both local dynamics and global semantics. Ablation studies confirm that this design choice yields the largest individual improvement (1.40%), validating that architectural alignment with linguistic structure enhances recognition.

The method’s cross-lingual success (97.1% Chinese, 97.07% Turkish) demonstrates that these principles transcend specific sign languages. The key insight is that bridging vision and language requires not just technical integration but theoretical alignment with how signs convey meaning through structured, multi-component expressions with natural variation. Future work should explore these principles in continuous signing and sign language translation tasks.

## Figures and Tables

**Table 1:** Alignment results comparing primary $T_{b}$ and refined $T_{g}$ descriptions, highlighting: sign label, substitute descriptions, expert-validated knowledge, primary descriptions *T_b_*, and refined descriptions $T_{g}$.

Primary Descriptions $T_{b}$	Expert-Validated Knowledge	Refined Descriptions $T_{g}$
Devoted: (I) Make the sign for “love”. (II) Extend the thumb with one hand and place it on the palm of the other hand, then raise it upwards.	The sign for “love”: Gently caress the back of the thumb with one hand, expressing a feeling of “tenderness”.	Devoted: (1) Gently caress the back of the thumb with one hand, expressing a feeling of “tenderness”. (2) Extend your thumb with one hand, sit on the other palm, and lift it up.
Ambience: (1) One hand forms the manual sign “Q”, with the fingertips pointing inward, placed at the nostrils. (2) Extend your index finger with one hand and make a big circle with your fingertips facing down.	The manual sign “Q”: One hand with the right thumb down, the index and middle fingers together on top, the thumb, index, and middle fingers pinched together, the fingertips pointing forward and slightly to the left, the ring and little fingers bent, the fingertips touching the palm.	Ambience: (1) One hand with the right thumb down, the index and middle fingers together on top, the thumb, index, and middle fingers pinched together, the fingertips pointing forward and slightly to the left, the ring and little fingers bent, the fingertips touching the palm. The fingertips are pointing inward, placed at the nostrils. (2) Extend index finger with one hand and make a circle with your fingertips facing down.

**Table 2:** Performance comparison on the SLR-500 dataset.

Method	Accuracy (%)
ST-GCN [34]	90.0
SignBERT [19]	94.5
BEST [7]	95.4
MASA [8]	96.3
SSRL [35]	96.9
Ours (joint\joint_motion)	96.01\95.28
Ours (bone\bone_motion)	94.37\94.19
Ours (4 streams fusion)	97.1

**Table 3:** Performance comparison on AUTSL dataset (Top-1 and Top-5 accuracy in %).

Method	Top-1	Top-5
SL-TSSI-DenseNet [36]	93.13	–
SSTCN [37]	93.37	–
SAM-SLR-V1 [37]	95.45	99.25
AM-GCN-A [38]	96.27	99.48
SAM-SLR-V2 [39]	96.47	99.76
TMS-Net [40]	96.62	99.71
SML [41]	96.85	99.79
Ours	97.07	99.89

**Table 4:** Comprehensive evaluation metrics on balanced SLR500 and AUTSL datasets.

Dataset	Accuracy	Precision	Recall	F1-Score	Top-5 Acc
SLR500	97.1%	96.5%	96.8%	96.7%	99.6%
AUTSL	97.07%	96.2%	96.8%	96.5%	99.89%

**Table 5:** Ablation study on the description generation components. VE: Visual Encoder, KB: Knowledge Base.

VE	LLM	KB	Optimized Prompt	Accuracy (%)
✓	–	–	–	93.85
✓	✓	–	–	93.57
✓	–	✓	–	94.50
✓	✓	✓	–	94.89
✓	✓	✓	✓	95.25

✓ Indicates that the component was included in the experimental setup.

**Table 6:** Ablation study of hierarchical multipart contrastive learning (based on joint data).

VE	Synonym	Prompt	Multipart	Accuracy (%)
✓	–	–	–	93.85
✓	✓	–	–	94.50
✓	–	✓	–	94.93
✓	–	–	✓	95.25
✓	✓	✓	–	95.38
✓	✓	✓	✓	95.81

✓ Indicates the inclusion of the corresponding element or knowledge source.

**Table 7:** Comparison of the effects of different keypoint combinations in SLR.

Method	Num. Keypoints	Acc (%)	Parts	+Multipart (%)
all	133	59.22	–	–
base	27	93.46	3	93.62 (0.16%↑)
MASA	49	93.66	3	94.01 (0.35%↑)
Cosign	76	93.57	5	94.52 (0.95%↑)
Ours	87	93.85	5	95.25(1.40%↑)

**Table 8:** Ablation study on the contribution of different body part features. The baseline is the Visual Encoder (VE) only, without multipart contrastive learning.

Configuration	Accuracy (%)	Performance Drop
VE-only (Baseline)	93.85	-
VE + Multipart (Full Model)	**95.25**	-
w/o Face features	94.55	−0.70
w/o Body features	95.04	−0.21
w/o Hands features	93.92	−1.33

**Table 9:** Computational cost analysis. Our method is compared with the baseline on an NVIDIA A100 GPU. The costs for our method are presented separately for the training and inference stages to provide a clear comparison.

Method	Training Time (h)	GPU Memory (GB)	Inference (ms/Sample)	Parameters (M)
CTR-GCN (Baseline)	8.2	12.4	4.2	5.6
Ours (Training)	21.3	24.6	-	69.1
Ours (Inference)	-	12.8	4.3	5.6

## Data Availability

The original SLR-500 and AUTSL datasets are publicly available from their respective sources (SLR-500: https://ustc-slr.github.io/datasets/2015_csl/ (accessed on 21 September 2025), AUTSL: https://cvml.ankara.edu.tr/datasets/ (accessed on 21 September 2025)). Our Supplementary Materials will be hosted at https://github.com/LiangSiyv/GSPMC (accessed on 21 September 2025).

## Supplementary Materials

The following supporting information can be downloaded at: https://github.com/LiangSiyv/GSPMC (accessed on 21 September 2025).
